# A Silent Threat Unveiled: Mycobacterium gordonae Catheter-Related Peritonitis in a Patient on Peritoneal Dialysis

**DOI:** 10.7759/cureus.76280

**Published:** 2024-12-23

**Authors:** Min Aung Hein, Uraiwan Parinyasiri, Dhammika Lehan Wannigama, Nibondh Udomsantisuk, Talerngsak Kanjanabuch

**Affiliations:** 1 Department of Nephrology, No. (1) 1000-Bedded Defence Services General Hospital, Yangon, MMR; 2 Kidney Diseases Clinic, Department of Internal Medicines, Songkhla Hospital, Songkhla, THA; 3 Department of Microbiology, Faculty of Medicine, Chulalongkorn University, Bangkok, THA; 4 Center of Excellence in Antimicrobial Resistance and Stewardship, Faculty of Medicine, Chulalongkorn University, Bangkok, THA; 5 Department of Infectious Diseases and Infection Control, Yamagata Prefectural Central Hospital, Yamagata, JPN; 6 School of Medicine, Faculty of Health and Medical Sciences, The University of Western Australia, Perth, AUS; 7 Biofilms and Antimicrobial Resistance Consortium of ODA Receiving Countries, The University of Sheffield, Sheffield, GBR; 8 Pathogen Hunter’s Research Collaborative Team, Department of Infectious Diseases and Infection Control, Yamagata Prefectural Central Hospital, Yamagata, JPN; 9 Division of Nephrology, Department of Medicine, Faculty of Medicine, Chulalongkorn University, Bangkok, THA; 10 Peritoneal Dialysis Excellent Center, King Chulalongkorn Memorial Hospital, Bangkok, THA; 11 Center of Excellence in Kidney Metabolic Disorders, Faculty of Medicine, Chulalongkorn University, Bangkok, THA

**Keywords:** catheter-related infection, exit site infection, mycobacterium gordonae, non-tuberculous mycobacterium, peritoneal dialysis, peritonitis

## Abstract

Infectious complications in peritoneal dialysis (PD) remain a constant challenge, with atypical pathogens posing significant risks. This case from Thailand highlights the rare occurrence of *Mycobacterium* *gordonae*, an often-overlooked non-tuberculous mycobacterium (NTM), as the causative agent in a catheter-related exit-site infection that progressed to peritonitis. Initially misattributed to *Pseudomonas aeruginosa* from preceding exit-site infections, *M. gordonae* was ultimately identified as the primary pathogen through multiple effluent cultures and advance polymerase chain reaction sequencing. This case underscores the importance of heightened clinical suspicion, early and accurate diagnosis, and timely interventions to prevent severe complications, including hemodialysis transfer.

## Introduction

Infectious complications pose a significant challenge in peritoneal dialysis (PD), often leading to unnecessary hemodialysis (HD) transfer and contributing to increased morbidity and mortality [1]. While most PD-related infections are caused by common Gram-positive or Gram-negative bacteria, the emergence of atypical infections caused by non-tuberculous mycobacteria (NTM) is becoming a growing concern [2]. These infections are particularly problematic due to their atypical presentation and the difficulties associated with their diagnosis and treatment. *Mycobacterium gordonae*, though rare, is a notable NTM pathogen in PD-related infections [3-4]. To date, only four cases of *M. gordonae* causing peritonitis or exit-site infection (ESI) have been reported globally, in the US and Japan. Of these, three required a transition to HD. Notably, none of these cases confirmed the species using polymerase chain reaction (PCR), raising questions about the accuracy of pathogen identification [3-6]. In this report, we present a rare case of refractory *M. gordonae* ESI that progressed to peritonitis in patients on PD, highlighting the critical importance of early and accurate diagnosis to prevent poor PD outcomes.

## Case presentation

A 47-year-old Thai woman with kidney failure due to type II diabetes mellitus and hypertension had been on continuous ambulatory PD (CAPD, 2 L x 4 exchanges/day) for four years. Her blood pressure, blood sugar levels, fluid balance, and nutritional status were all well-maintained, with no history of PD-related complications. However, four months before this presentation, she developed pus discharge and erythema at the exit site, leading to the diagnosis of ESI. A Gram stain of the discharge revealed a mixed infection, with culture identifying *Pseudomonas aeruginosa* and an initially unidentified Gram-positive organism, later confirmed to be *M. gordonae*. She was treated with oral ciprofloxacin 500 mg daily for three weeks, following the 2023 International Society for Peritoneal Dialysis (ISPD) Catheter-related Infection Guidelines and antimicrobial susceptibility results for *P. aeruginosa*, which led to partial resolution of the infection [7].

One month later, she experienced a second episode of ESI, this time involving only Gram-positive bacilli. Despite treatment with a repeat antibiotic regimen, the infection partially resolved. A closer review of her PD practices uncovered lapses in aseptic technique, such as incomplete hand disinfection and rushed exchange procedures. She was subsequently retrained on proper exit-site care and securing the PD catheter to prevent traction or torsion trauma.

**Figure 1:**
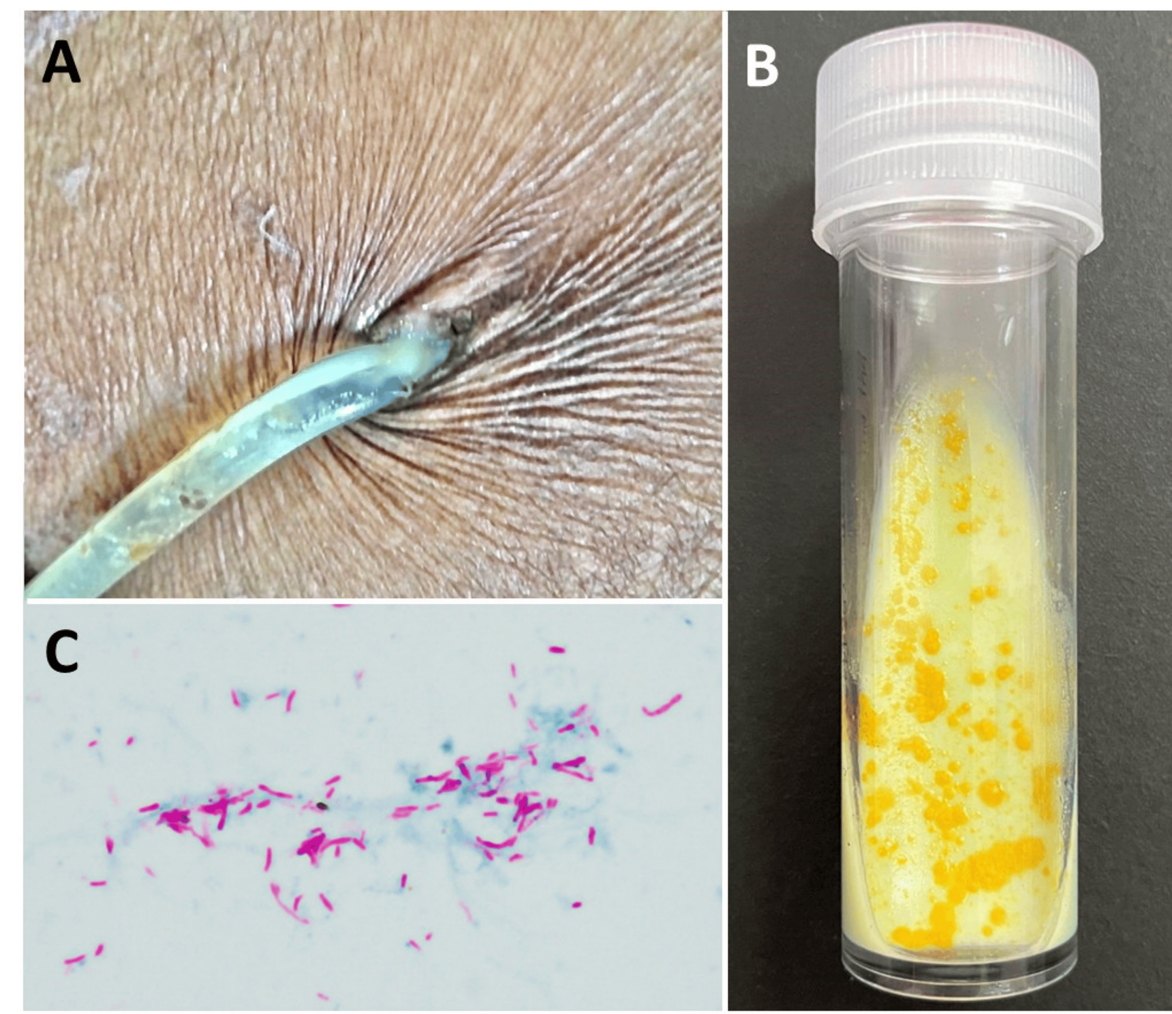
Clinical and Microbiological Findings in a Case of Chronic Mycobacterium gordonae Catheter-Related Peritonitis (A) Chronic exit-site infection with yellowish discharge and moderate crusting surrounding the peritoneal dialysis catheter. (B) *M. gordonae* colony on Loewenstein-Jensen medium (LJ) (Biomedia, Thailand), showing its distinct yellow-orange pigmentation. (C) Acid-fast bacilli visualized by AFB staining, indicating the presence of mycobacteria.

On March 24, 2024 (Day 0), the patient presented with cloudy PD effluent (PDE), abdominal pain, and diarrhea. Examination of the exit site revealed signs of chronic ESI, including mild inflammation, granulation, and yellowish discharge with moderate crusting (Figure 1A). Ultrasonography of the tunnel tract showed no collection around the PD catheter. Samples of PDE and an exit-site swab were submitted for Gram stain, cell count, and cultures, including fungal and mycobacterial cultures. Initial PDE analysis revealed a PDE leukocyte count of 5,035 cell/mm^3^ with 95% neutrophils-clearly meeting the 2022 ISPD Peritonitis Guidelines for the diagnosis of PD-related peritonitis, defined as an effluent cell count >100 cell/mm^3^ with neutrophil dominance [8].

The patient was treated with intraperitoneal (IP) meropenem 1 gm and gentamicin 40 mg once daily for suspected catheter-related peritonitis due to *P. aeruginosa*. While her symptoms improved, with clear PDE and a reduced leukocyte count (3,665 cell/mm^3^, 98% neutrophils), persistent effluent leukocytosis (>100 cell/mm^3^) on day 5 despite appropriate antibiotic therapy indicated refractory peritonitis, as defined by the 2022 ISPD Peritonitis Guidelines [8]. As a result, her PD catheter was removed, and she was transitioned to HD twice weekly. Post-catheter removal, intravenous gentamicin therapy was continued for a total of 14 days to ensure optimal management of her condition.

PCR testing of the PDEs and pus from the tunnel tract confirmed mycobacteria, diagnosing a PD catheter-related infection according to the 2022 ISPD Peritonitis Guidelines [8]. Myco-F/Myco-R primers revealed 99-100% coverage with 98.1-98.5% identity, while sequencing with UFUL/URUL primers identified *M. gordonae*, with 98-100% coverage and 96.4-97.1% identity (MK890518.1). These findings were confirmed by culture, establishing *M. gordonae* as the causative agent (Figure 1B-1C). No growth of *P. aeruginosa* was observed in multiple PDE cultures, ruling out coinfection. Antimicrobial susceptibility testing revealed that the isolate was susceptible to amikacin, clarithromycin, linezolid, moxifloxacin, and trimethoprim/sulfamethoxazole, while resistant to ciprofloxacin, doxycycline, and rifampin. Intermediate susceptibility was noted for ethambutol and rifabutin, with no interpretation available for ethionamide, isoniazid, and streptomycin. Based on these results, the patient was treated with trimethoprim 160 mg/sulfamethoxazole 800 mg twice daily, azithromycin 500 mg three times a week, and levofloxacin 500 mg once daily for six months. The patient was later resumed PD without recurrence of peritonitis.

## Discussion

This case involves a 47-year-old woman on CAPD who developed an ESI initially attributed to *P. aeruginosa*, with *Mycobacterium* mistakenly dismissed as a contaminant. The infection partially subsided but relapsed a month later, and the *Mycobacterium* was repeatedly overlooked. After four months, the undiagnosed *M. gordonae* caused PD-catheter peritonitis, leading to PD termination and transfer to HD.

In addition to retraining on exit-site dressing and aseptic techniques, refractory ESIs should raise suspicion for atypical organisms like NTM or fungi. Rapidly growing NTMs, such as *Mycobacterium abscessus*, are often misidentified as diphtheroids or *Corynebacterium* species, causing delayed or missed diagnoses. When NTM infection is suspected, testing for acid-fast bacilli with Ziehl-Neelsen staining and culturing on specialized media is critical [9]. While most NTM infections in PD patients are caused by rapidly growing species like *Mycobacterium ​fortuitum*, *M. abscessus*, and *Mycobacterium chelonae* [10-13], infections with slower-growing NTMs, such as *M. gordonae* are relatively rare (Table 1). Notably, no previously reported cases of *M. gordonae* in PD patients have presented with catheter-related peritonitis.

**Table 1:** Published Case Reports of Mycobacterium gordonae in Patients on Peritoneal Dialysis APD, Automated Peritoneal Dialysis; CAPD, Continuous Ambulatory Peritoneal Dialysis; CGN, Chronic Glomerulonephritis; HD, Hemodialysis; IVDU, Intravenous Drug User; PD, Peritoneal Dialysis

Case	Country	Peritonitis	ESI	Tunnel infection	Treatment	Duration	Catheter removal	Outcomes	Ref.
36-Y male, CAPD, IVDU	United States	Yes	No	No	Amikacin, Pyrazinamide	7 M	Yes	HD	London et al. [3]
39-Y female, CGN, CAPD for 5 Y	United States	Yes	No	No	Isoniazid, Ethambutol, Rifampicin, Amikacin, Clarithromycin	18 M	Yes	HD, not favorable outcome	Harro et al. [4]
62-Y female, with Takayasu arteritis, APD for 3 M	Japan	No	Yes	Yes	Levofloxacin, Clarithromycin, Ethambutol	7 W	Partial reimplantation	PD	Hirohama et al. [5]
54-Y female, CAPD for 5 Y	United States	Yes	No	No	Isoniazid, Rifampicin, Pyrazinamide, Ethambutol	3 M	Yes	HD	Asnis et al. [6]
47-Y female, diabetes, CAPD for 4 Y	Thailand	Yes	Yes	Yes	Bactrim, Levofloxacin, Azithromycin	7 W	Yes	PD	Our presented case

*M. gordonae*, commonly found in environmental and clinical settings, is generally considered nonpathogenic and is the most frequently isolated mycobacterial contaminant. Although only 23 clinically significant cases were confirmed before 1992, *M. gordonae* can occasionally cause infections, particularly in immune-compromised individuals [14-18]. To confirm a clinically significant infection, specific criteria proposed by Weinberger [14] are recommended: (1) multiple isolations from the same or different body sites; (2) detection in smears or histologic sections; (3) growth from a single specimen on multiple media; (4) presence of a clinical illness or histopathological process consistent with mycobacterial infection; (5) response to appropriate antimycobacterial therapy; and (6) elimination of the organism as the clinical condition improves. This case met all these criteria, confirming *M. gordonae* as the causative pathogen.

The 2022 ISPD Peritonitis Guidelines recommend a combination of effective antibiotics and catheter removal (2D) for treatment [8]. While the guidelines do not specify the type or duration of antimycobacterial therapy, expert opinion suggests using two susceptible agents for at least six weeks. Similarly, the Infectious Diseases Society of America (IDSA) advises removing any foreign bodies to enhance treatment success [11]. In our case, successful treatment was achieved through catheter removal and antimycobacterial therapy, in line with both the ISPD and IDSA recommendations.

## Conclusions

In conclusion, this case underscores the need for a high index of suspicion for atypical organisms like NTM in refractory or culture-negative infections. Delayed or missed identification of the causative organism can result in refractory infections, complications, and poor outcomes. Early recognition of *M. gordonae *could have avoided the temporary transition to HD in this patient.
